# Advances in targeting protein S-palmitoylation in tumor immunity and therapy

**DOI:** 10.3389/fonc.2025.1547636

**Published:** 2025-02-24

**Authors:** Miaomiao Han, Yuanhao Lv, Yiyang Chen, Zhaoyi Li, Jiaqi Tian, Hongyan Zhou, Yunlong Wang, Wei Su, Jiateng Zhong

**Affiliations:** ^1^ Department of Pathology, School of Basic Medical Sciences, Xinxiang Medical University, Xinxiang, China; ^2^ Department of Pathology, The First Affiliated Hospital of Xinxiang Medical University, Xinxiang, China; ^3^ Henan Bioengineering Technology Research Center, Zhengzhou, China; ^4^ Xinxiang Key Laboratory of Precision Diagnosis and Treatment for Colorectal Cancer, Xinxiang First People’s Hospital, Xinxiang, China; ^5^ Xinxiang Engineering Technology Research Center of Digestive Tumor Molecular Diagnosis, The First Affiliated Hospital of Xinxiang Medical University, Xinxiang, China

**Keywords:** S-palmitoylation, tumor, immunotherapy, T cells, immune escape, drug resistance

## Abstract

S-palmitoylation is a reversible and dynamic post-translational modification of proteins. A palmitoyl group is covalently attached to a cysteine residue of the protein by a thioester link. It regulates the transcription and expression of downstream target genes and cell signaling, influencing cellular functions. Research indicates a substantial correlation between S-palmitoylation and tumorigenesis and immunotherapy, where it plays a pivotal role in modulating T cell activation, cytokine signaling, autophagy, phagocytosis, and death. Moreover, palmitoylation contributes to drug resistance and immunological evasion in tumor cells, enabling them to circumvent the effects of chemotherapeutic drugs and immune surveillance. Inhibitors that target S-palmitoylation have demonstrated significant potential in enhancing the efficacy of tumor immunotherapy, offering a novel strategy for cancer treatment. Nonetheless, obstacles such as inhibitor specificity and efficacy persist, requiring more extensive investigations into the exact mechanisms of S-palmitoylation to develop more effective targeted therapeutics. This article summarizes recent developments in S-palmitoylation concerning tumor immunity and treatment. The article examines the regulatory function of S-palmitoylation, its modifying enzymes in tumor cell signaling, and novel tumor immunotherapies that target S-palmitoylation.

## 1 Introduction

Immunotherapy introduces a novel approach to cancer treatment by enhancing and empowering the body’s immune system, allowing it to identify and eliminate tumor cells effectively. Many clinical studies have demonstrated that immunotherapy exhibits impressive effectiveness in treating various malignant tumors, including melanoma, non-small cell lung cancer, and renal cell carcinoma (1). This approach significantly extends patient survival and substantially decreases the disease’s recurrence rate, offering hope to countless individuals! (2).

Meanwhile, lipidation modification, an essential mode of protein post-translational modification, allows lipoproteins to play a pivotal role in intracellular localization, translocation, protein-protein interactions, and stability due to their special affinity for the phospholipid bilayer (3). S-palmitoylation, first identified in the 1980s, is a highly conserved post-translational modification of proteins found in all eukaryotic organisms (4). Palmitoyl transferase enzymes (PATs) catalyze a process in which the palmitoyl group (C16:0) is covalently attached to the sulfhydryl group of the protein’s C-terminal cysteine (Cys) through a thioester bond. This reversible modification allows for studying its effects on protein function *in vitro*. Additionally, it plays a significant role in regulating protein subcellular localization, enzyme activity, stability, and protein interactions across various aspects. S-palmitoylation is, therefore, essential in complex physiopathological processes, including cell signaling and the development of diseases (5, 6).

S-palmitoylation also plays a vital role in immunomodulation. It governs the activity and function of immune cells, thereby influencing the body’s immune response. This discovery certainly offers fresh concepts and targets for tumor immunotherapy. This paper presents the process of S-palmitoylation, detailing its role in immune regulation and exploring the potential of targeting S-palmitoylation for tumor immunotherapy.

## 2 Protein palmitoylation regulation process

**Figure 1:**
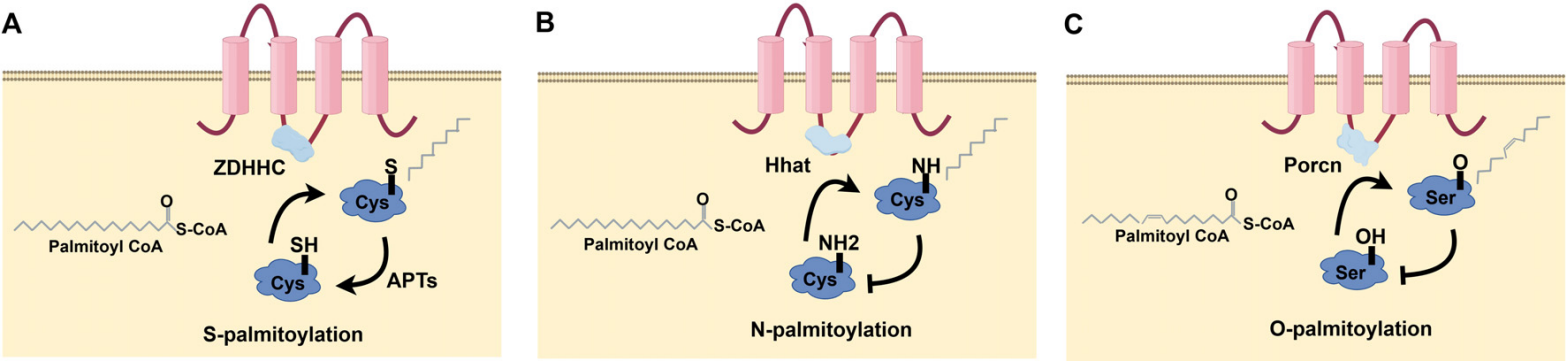
Types of protein palmitoylation: **(A)** S-palmitoylation, **(B)** N-palmitoylation, **(C)** O-palmitoylation.

Protein palmitoylation changes arise from alterations to cysteine side chains and are categorized into three kinds according to their connection (**Figure 1**): S-palmitoylation is the process whereby a long-chain fatty acid, typically palmitic acid (16 carbons), covalently attaches to a Cys residue in a protein, resulting in an unstable thioester bond. This dynamic and reversible post-translational modification is prevalent across organisms. It plays crucial roles in regulating protein structure, transport, cellular localization, stability, and interactions and participating in numerous biological processes. It is implicated in multiple biological processes and intimately associated with various illnesses’ emergence and progression (**Figure 1A**). N-palmitoylation denotes the conjugation of palmitic acid to a glycine (Gly) or Cys residue of a protein through an amide link. While less prevalent than S-palmitoylation, this variant is seen in specific proteins (**Figure 1B**). O-palmitoylation denotes the conjugation of palmitic acid to the hydroxyl group of a serine (Ser) or threonine (Thr) residue inside a protein. This alteration is uncommon but may be significant in some particular proteins (**Figure 1C**). Consequently, the palmitoylation alteration is commonly designated as S-palmitoylation. Palmitoylation is the most common of all lipidation changes, influencing over 20% of the proteome (7).

S-palmitoylation is a dynamic process that modulates protein biological properties over seconds to hours. This process is governed by PATs and acylprotein thioesterases (APTs), with PATs being pivotal in protein palmitoylation (8). In mammals, PATs predominantly comprise members of the zinc finger DHHC-type family (ZDHHC), which includes 23 distinct proteins (ZDHHC1-23) (9). Most are in the endoplasmic reticulum or Golgi membrane, with ZDHHC5, 20, and 21 in the plasma membrane (10, 11). ZDHHCs facilitate protein palmitoylation via a two-step mechanism, first with self-auto palmitoylation to create an acyl-enzyme intermediate, subsequently transferring acyl-coenzyme A to cysteine residues in the substrate protein (12). Protein depalmitoylation removes thioester-linked long-chain fatty acids from cysteine residues in proteins. Acyl protein thioesterases catalyze thioester hydrolysis for numerous S-palmitoylated proteins, therefore solubilizing and displacing membrane substrate proteins, aided by acyl protein thioesterases (13). Compared to the countless PATs, only a few depalmitoylating enzyme classes have been thoroughly investigated, namely APT1, APT2, PPT1, PPT2, and ABHD17 (14, 15).

## 3 S-palmitoylation and the immunological response

**Figure 2:**
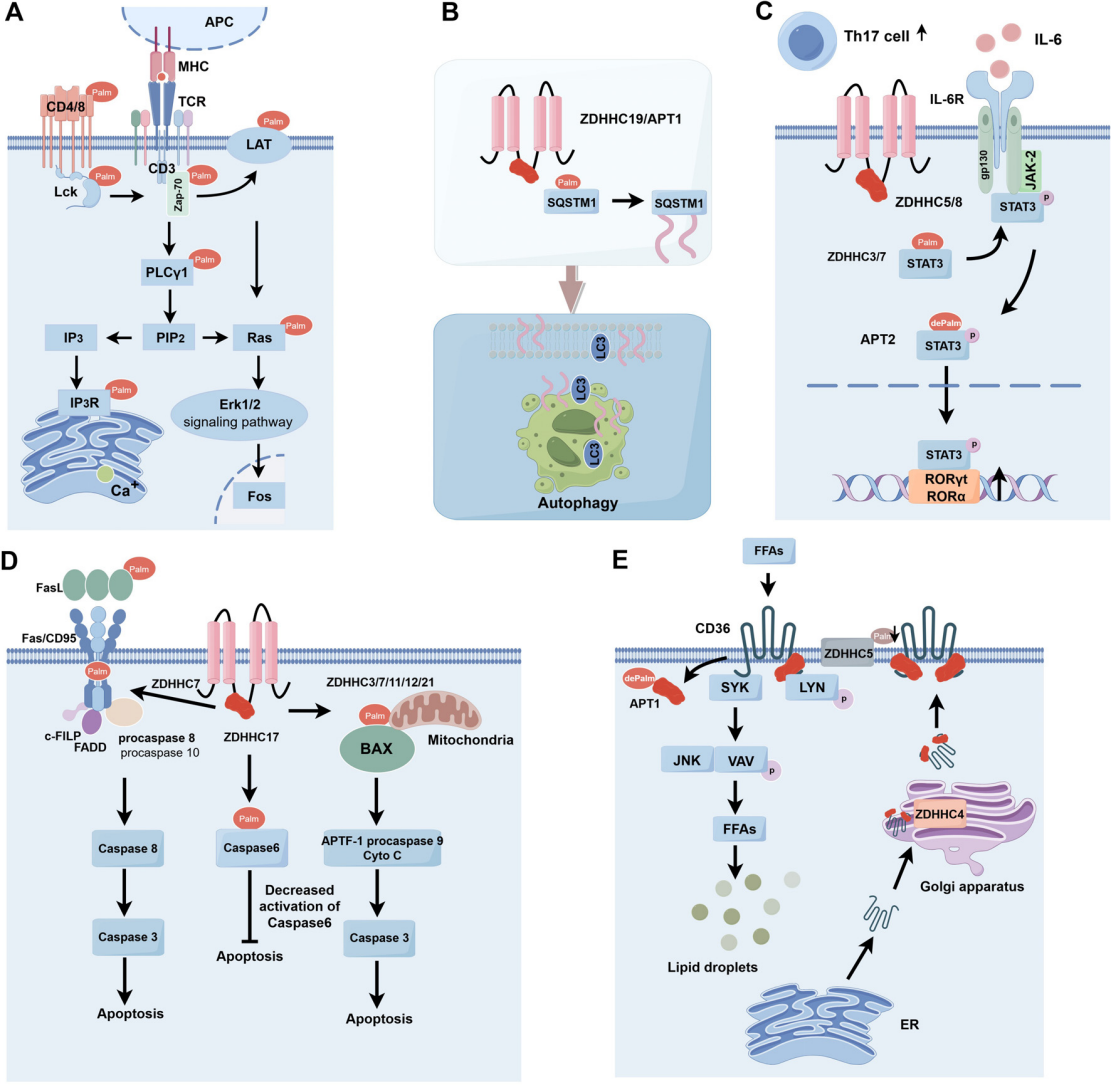
S-palmitoylation is involved in immune response types. **(A)** S-palmitoylation plays a key role in the modification of TCR signaling pathway-associated proteins (e.g. CD4, CD8, Lck, Zap-70, LAT, and PLCγ1), affecting their membrane localization, stability, and signaling, and thus regulating T cell activation and function. **(B)** ZDHHC19/APT1 promotes the localization of SQSTM1/P62 on phagocyte membranes by regulating its S-palmitoylation and synergizes with LC3 for autophagosome formation and pathogen isolation. **(C)** Enzymes such as ZDHHC5/8/7/3 are involved in the regulation of Gp130 and STAT3 in the IL-6 signaling pathway through S-palmitoylation modification, which affects their plasma membrane localization and phosphorylation status, and thus promotes STAT3 activation and nuclear translocation, and ultimately regulates the differentiation of Th17 cells. **(D)** Enzymes such as ZDHHC3/7/11/12/21 play important roles in apoptosis of immune and tumor cells by regulating the Fas/FasL signaling pathway, cysteine asparaginase activity, and the function of Bcl-2 family proteins. **(E)** Enzymes such as ZDHHC5 and APT1 regulate the dynamic palmitoylation process of CD36, which affects lipid recognition and phagocytosis and is critical for fatty acid uptake and immune cell function. APC, Antigen Presenting Cell; MHC, Major Histocompatibility Complex; TCR, T Cell Receptor; Lck, Lymphocyte-specific protein tyrosine kinase; LAT, Linker for Activation of T cells; Zap-70, Zeta-chain-associated protein kinase 70; PLCg1, Phospholipase C gamma 1; PIP2, Phosphatidylinositol (4,5) bisphosphate; IP3, Inositol triphosphate; IP3R, Inositol 1,4,5-trisphosphate (IP3) receptor; Ras, Rat sarcoma; Erk1/2, Extracellular regulated protein kinases; SQSTM1, Sequestosome 1; LC3, Microtubule-associated protein 1 light chain 3;Th17 cell, T helper cell 17; IL-6, Interleukin-6; IL-6R, Interleukin 6 Receptor; gp130, Glycoprotein 130; JAK-2, Janus kinase 2; STAT3, Signal Transducer and Activator of Transcription 3; FasL, Fas ligand; Fas/CD95, Factor-related Apoptosis; c-FLIP, cellular FLICE-inhibitory protein; FADD, Fas-associating protein with a novel death domain; APAF-1, Apoptotic protease-activating factor-1; FFAs, Free Fatty Acids; SYK, Spleen tyrosine kinase; LYN, Lck/Yesrelated novel protein tyrosine kinase; JNK, c-Jun N-terminal kinase; VAV, Vav guanine nucleotide exchange factor; ER, Estrogen Receptor.

S-palmitoylation, a significant post-translational modification of proteins, is crucial in carcinogenesis and progression by modifying protein characteristics and functions and has surfaced as a potential therapeutic target. S-palmitoylation significantly influences the functionality of specific essential proteins, particularly those involved in immune cell signaling and activation. S-palmitoylation can influence proteins inside the T-cell receptor (TCR) complex, consequently modulating T-cell activation and functionality (16). S-palmitoylation can affect cytokine receptors’ functionality, influencing immune cell responses and mediation (**Figure 2**).

### 3.1 S-palmitoylation alters proteins associated with the TCR signaling pathway

T cells are crucial in the adaptive immune response, mediating antigen-specific cellular immunity, aiding B cells in antibody generation, and facilitating the activity of other immune system cells. The development and activation of T cells require TCR signaling (17). S-palmitoylation is essential for regulating immunological responses and plays a role in T-cell activation and signal transmission (18). Upon the binding of an antigen to a major histocompatibility complex (MHC) molecule, an antigen-MHC complex is established, which is then recognized by the TCR, leading to its activation and aggregation to create an immunological synapse. Upon binding of the TCR to the antigen-MHC complex, phosphorylation of the immunoreceptor tyrosine-based activation motif (ITAM) occurs inside the cytoplasmic structural domain of the CD3 subunit, subsequently initiating the signaling cascade via activated tyrosine kinase (19). CD45 receptor tyrosine phosphatases modulate the phosphorylation and activation of Lck and other Src family tyrosine kinases (20). Zeta-chain-associated protein kinase (Zap-70) is recruited to the TCR/CD3 complex, where it aggregates and activates, subsequently facilitating the recruitment and phosphorylation of downstream junctional or backbone proteins. This process enables T cells to recognize and respond to antigens via TCR signaling, thereby playing a crucial role in the regulation and execution of the immune response, including responses to infections and tumors (**Figure 2A**) (21).

#### 3.1.1 CD4 and CD8

CD4 and CD8 are present on the surface of T lymphocytes. Initially, they possess dual capabilities as TCR co-receptors that bind to MHC class II and class I molecules, thus facilitating TCR recognition of MHC-bound peptide antigens by enhancing T cell-antigen-presenting cell interactions. Secondly, the cytoplasmic tails of both co-receptors interact with Lck tyrosine kinase, facilitating its activation and functional integration with the TCR signaling machinery (22). CD4 undergoes palmitoylation at two membrane-proximal cysteine residues, Cys396 and Cys399 (23), while CD8 on T cells comprises disulfide-linked CD8α and CD8β chains, which are transmembrane proteins (24). CD4 and CD8αβ are both situated within membrane lipid rafts. The palmitoylation of CD4, in association with Lck, enhances the accumulation of CD4 in lipid rafts (25). The presence of CD4 in lipid rafts is associated with its capacity to augment receptor tyrosine phosphorylation. On the contrary, in mice, only CD8β undergoes palmitoylation. Palmitoylation of Mouse CD8β is essential for optimal CD8 co-receptor functionality since it enhances CD8’s interaction with Lck within lipid rafts (26).

#### 3.1.2 Lck

Lck is palmitoylated at Cys3 and Cys5 (27). Palmitoylation of Lck is non-essential for its catalytic activity but crucial for its localization to the plasma membrane, facilitating TCR signaling and enabling T cell activation (28, 29). Lck is recognized to undergo palmitoylation at Cys3 and Cys5 (27). This lipid modification is essential for Lck to facilitate its membrane localization and association with lipid rafts (27, 30, 31). Inhibition of the palmitoylation site of Lck obstructs its association with the membrane and lipid rafts, impairing its cellular functions (31, 32). Both protein acyltransferases ZDHHC2 and ZDHHC21 have been reported to specifically palmitoylate Lck at the plasma membrane (33, 34). The impaired expression of ZDHHC21 did not influence the overall protein expression level; however, it inhibited the S-palmitoylation of crucial T-cell signaling proteins, resulting in a marked decrease in the palmitoylation of Lck and Fyn, a decline in the phosphorylation of several essential signaling proteins, and a reduction in the expression of T-cell activation markers (34).

#### 2.1.3 Zap-70

Zap-70, a non-receptor tyrosine kinase, is essential for the immune response of T cells. In Zap-70-deficient T cells, the phosphorylation of LAT and SLP-76 is compromised, the formation of the LAT signaling protein complex is obstructed, and the activation of downstream signaling pathways is hindered (35). Zap-70 has been shown to experience TCR-dependent palmitoylation at the kinase structural domain Cys564 (36). OKT3 stimulation of T cells results in enhanced S-palmitoylation of Zap-70; however, palmitoylation does not influence the plasma membrane localization or stability of Zap-70, nor is it necessary for its kinase activity, but it is essential to its interaction with substrates and for the transduction of TCR signals. Cys564 acylation-deficient mutants of Zap-70 cannot phosphorylate LAT and SLP-76, leading to the breakdown of the TCR signaling pathway and a marked decrease in T cell activation and production of T cell surface markers (36).

#### 3.1.4 LAT

LAT is a key junctional molecule that modulates T cell growth and functionality. It is situated at the plasma membrane and conveys proximal signals initiated by TCR stimulation, hence playing an essential role in T cell activation (37). Phosphorylated LAT binds and activates growth factor receptor-binding protein 2 (GRB2), phospholipase Cγ1 (PLCγ1), phosphatidylinositol 3-kinase (PI3K), and additional signaling molecules, leading to the transmission of signals essential for T cell activation and function. LAT undergoes palmitoylation at the Cys26 and Cys29 sites at the interface of its transmembrane and cytoplasmic structural domains (38). The palmitoylation of the LAT transmembrane structural domain and the Cys26 site is crucial for its stability and localization inside lipid rafts; the absence of LAT palmitoylation led to a marked reduction in LAT production and impeded its localization in lipid rafts (39, 40). Moreover, LAT mutants exhibited deficient recruitment of PLCγ1 and could not transmit TCR-mediated signaling (41, 42). In antigen-specific incompetent T cells, significantly compromised LAT palmitoylation resulted in a marked decrease in LAT recruitment at the immunological synapse and inhibited TCR/CD28-induced phosphorylation, along with subsequent activation of PLCγ1, indicating that the dynamic palmitoylation of LAT plays a role in T cell incompetence (43).

#### 3.1.5 PLCγ1

Calcium ion endocytosis serves as an essential marker of T cell activation (44). PLCγ1, an essential protein in the calcium signaling cascade, is activated by Itk to hydrolyze phosphatidylinositide 4,5-bisphosphate (PIP2) into diacylglycerol (DAG) and inositol 1,4,5-trisphosphate (IP3). IP3 interacts with its receptor IP3R on the endoplasmic reticulum membrane, triggering the release of Ca2+ from the endoplasmic reticulum.

PLCγ1 has been reported to undergo palmitoylation by ZDHHC21 and is subject to dynamic regulation by TCR signaling (34). IP3R may undergo palmitoylation by ZDHHC6, with Cys56, Cys849, and Cys2214 identified as possible sites for this modification. Palmitoylation enhances the stability of IP3R protein production, while ZDHHC6 knockdown leads to diminished IP3R protein levels and a reduction in Ca2+ influx. The precise impacts of palmitoylation on PLCγ1 and IP3R proteins and the regulatory mechanisms in calcium signaling require more study (45).

### 3.2 Regulation of the autophagy process through S-palmitoylation

Autophagy can modulate antigen processing and presentation effectiveness, influencing T-cell activation and establishing immunological memory. Cells can utilize the autophagy route to convey internally synthesized antigens to MHC molecules, activating T cells and engaging in specific immunological responses (46, 47). SQSTM1, or P62, is a multifunctional protein that plays a vital role in immunity and cellular autophagy.SQSTM1/P62 was initially identified as an autophagy junction consisting of the PB1/TRAF6 binding domain (TB)/LC3 interacting region (LIR)/ubiquitin-associated (UBA) structural domains, which are involved in autophagy and apoptosis in tumor cells (48). CRCs expressing SQSTM1/P62 have been reported to modulate immunosuppressive Foxp3 regulatory T cells inside the tumor microenvironment (49). SQSTM1 is subjected to S-palmitoylation at the Cys289 and Cys290 residues, a process modulated by the protein acyltransferase ZDHHC19 and the acylprotein thioesterase LYPLA1/APT1. Upon activation of autophagy, S-palmitoylation of SQSTM1 secures SQSTM1 droplets to the phagocytic cell membrane. S-palmitoylation facilitates the LC3-SQSTM1 association, promoting the extension of the phagocyte membrane over the SQSTM1 droplet, which is subsequently encapsulated within the autophagosome (**Figure 2B**) (50).

Nucleotide-binding oligomerization domain-like receptor protein 3(NLRP3), an essential element of inflammatory vesicles, serves as a pivotal pattern recognition receptor that detects various microbial infections and endogenous danger signals, leading to the creation of inflammatory vesicles that activate caspase-1, resulting in the secretion of IL-1β and IL-18, as well as pyroptosis (51). S-palmitoylation of NLRP3 at the Cys844 location is essential for inhibiting inflammatory vesicle activation, and ZDHHC12 has been identified as the protein acyltransferase responsible for NLRP3 palmitoylation, facilitating its destruction via the chaperone-mediated autophagy pathway. Following inflammasome activation, ZDHHC12 expression is upregulated, facilitating NLRP3 degradation through the chaperone-mediated autophagy pathway, thus establishing a negative feedback loop that inhibits excessive activation of the NLRP3 inflammasome. Regulated activation of the NLRP3 inflammasome is crucial for immunological homeostasis (52, 53).

### 3.3 Regulation of cytokine receptor-mediated signaling through S-palmitoylation

Cytokines, including interleukins and interferons, are pivotal in tumor immune signaling. S-palmitoylation regulates many cytokine-mediated signaling pathways.

Interleukin-6 (IL-6) facilitates the targeted development of naïve CD4 T cells, therefore serving a crucial function in the interplay between innate and adaptive immune responses (54). It was shown that IL-6 binding to transforming growth factor (TGF)-β is essential for Th17 differentiation of naïve CD4 T cells (55). IL-6 signals through two proteins, IL6R and gp130, and activates signal transducer and activator of transcription 3 (STAT3) through Janus kinase2 (JAK2). Recent studies have shown that several proteins within the IL-6 signaling cascade are modulated by S-palmitoylation. The silencing of ZDHHC5 and ZDHHC8 is reported to influence gp130 location and subsequent STAT3 phosphorylation (56). STAT3 is regulated by a cycle of palmitoylation and depalmitoylation. ZDHHC7 and ZDHHC3 can palmitoylate STAT3 at the Cys108 location and migrate to the plasma membrane where gp130 and JAK2 are situated, facilitating STAT3 phosphorylation (57). Phosphorylated STAT3 can subsequently undergo depalmitoylation by APT2, promoting its translocation to the nucleus for gene induction, which is essential in Th17 cell development. Consequently, the palmitoylation cycle and depalmitoylation facilitate STAT3 activation and Th17 cell development. Th17 cells participate in numerous autoimmune disorders, such as inflammatory bowel disease, and the inhibition of palmitoylation (by ZDHHC7 knockdown) or depalmitoylation (via APT2 inhibition) would obstruct STAT3 activation and confer protection in a murine model of colitis. This research emphasizes the promise of targeting palmitoylation in treating autoimmune disorders (**Figure 2C**) (58).

### 3.4 Regulation of apoptotic signaling through S-palmitoylation

The immune system protects the body against pathogens and aberrant cells by identifying and eliminating apoptotic cells and modulating the intensity and duration of the immunological response. Recent findings indicate that S-palmitoylation of apoptosis-related proteins is disrupted in numerous human cancers (59). The TNF receptor family member Fas (CD95) assembles the death-inducing signaling complex (DISC) via the interaction of its death domain (DD) with the Fas ligand (FasL) (60). DISC comprises the Fas-associated death domain protein (FADD), the caspase regulator c-FLIP, and cysteinyl asparagine-8 (caspase-8); caspase-8 is cleaved into active heterotetramers and released from DISC, whereas activated caspase-8 triggers the apoptotic pathway (61). Cross-linking Fas with agonistic antibodies or FasL increases the active form caspase-8, hence inducing apoptosis (62). Fas/FasL is exposed to many post-translational changes, with protein palmitoylation essential for regulating Fas/FasL signaling (63, 64). Fas can be S-palmitoylated at Cys199, a modification crucial for its location within lipid rafts and facilitating apoptotic actions (65). The S-palmitoylation of Fas, mediated by ZDHHC7, enhances the active form of cysteine-8 and promotes apoptosis (64). The ligand for the death receptor is controlled by palmitoylation. FasL can be S-palmitoylated at Cys82, facilitating its localization to lipid rafts and triggering apoptosis (66). Palmitoylation may also influence Fas downstream signaling pathways. ZDHHC17 has been documented to impede caspase-6 activation in neurons following S-palmitoylation of caspase-6 at the Cys264 and Cys277 residues (67). In addition, S-palmitoylation may also take place in proapoptotic members of the Bcl-2 family. The overexpression of various ZDHHCs, specifically ZDHHC3, 7, 11, and 12, enhances the S-palmitoylation of Bax and promotes apoptosis. The S-palmitoylation of Bax at the Cys126 site is essential for initiating apoptosis, as it influences its localization to the mitochondria (68). The Cys126 mutation markedly decreased the quantity of apoptotic cells and the activation of caspase-3 (68). These findings indicate that S-palmitoylation is crucial to the death of immunological and malignant cells (**Figure 2D**).

### 3.5 Regulation of phagocytosis through S-palmitoylation

Phagocytosis by immune cells initiates with identifying and attaching pathogens or foreign entities, typically facilitated by receptors on the phagocyte’s surface. Thereafter, the cell encloses the pathogen within the cell membrane to create phagocytic vesicles, which are subsequently sent to the lysosomes in the cytoplasm for destruction. The efficient operation of the immune system relies on the appropriate execution of phagocytosis, which is governed and synchronized by other immune system elements (69).

CD36 is a scavenger receptor that plays a critical role in immunity, metabolism, and several physiological functions (70). It identifies particular oxidized phospholipids, lipoproteins, and free fatty acids, facilitating phagocytosis and signal transduction mechanisms associated with these lipids. CD36 is modulated by a complicated cycle of palmitoylation and depalmitoylation (71). ZDHHC5 can maintain the palmitoylation of CD36 at the plasma membrane (72). CD36 can bind fatty acids, activate the LYN signaling pathway, and phosphorylate Y91 to decrease ZDHHC5 function. CD36 undergoes depalmitoylation by APT1, subsequently recruiting the tyrosine kinase SYK to phosphorylate JNK and VAV, initiating the endocytosis of fatty acid intake. CD36 undergoes palmitoylation by ZDHHC4 within the Golgi, facilitating translocation from the plasma membrane. Dynamic palmitoylation of CD36 significantly influences fatty acid absorption (**Figure 2E**) (73).

## 4 S-palmitoylation regulates tumor growth and metastasis

Significant cancer-related proteins undergo S-palmitoylation, which is closely linked to carcinogenesis and tumor growth (74, 75). The stimulation of 17β-estradiol (E2) enhances S-palmitoylation-dependent membrane localization of Estrogen Receptor beta (ERβ) and its interaction with Caveolin-1 and p38, ultimately promoting apoptosis in human colon adenocarcinoma DLD-1 cells via p38/MAPK pathway activation (76). S-palmitoylation of ERβ or suppressing p38/MAPK signaling has been documented to enhance colorectal cancer cell proliferation (77). Additionally, Wnt signaling is pivotal in carcinogenesis, particularly in CRC advancement (78). Wnt2B was demonstrated to be S-palmitoylated, and this modification influenced its cellular location, thus impacting Wnt signaling. Moreover, the levels of Wnt2B S-palmitoylation in mitochondria negatively correlated with intestinal tumorigenesis (79). Research indicates that the overexpression of cytoskeleton-associated protein 4 (CKAP4) or LDL receptor-related protein 6 (LRP6) facilitates the development of pancreatic cancer (80). CKAP4 was identified as S-palmitoylated by ZDHHC2 at the Cys100 position, while LRP6 was S-palmitoylated at the Cys1394 and Cys1399 sites. S-palmitoylation promotes the location of CKAP4 and LRP6 within detergent-resistant membrane (DRM) fractions, activating the PI3K-AKT pathway and enhancing pancreatic cancer cell proliferation (81). ZDHHC12 facilitates the S-palmitoylation of claudin3 at the Cys103, Cys106, Cys181, Cys182, and Cys184 positions, promoting ovarian cancer progression (82). S-palmitoylation of phosphatidylinositol 4-kinase II alpha (PI4KII alpha) has enhanced mouse tumor growth by altering its catalytic activity and subcellular location (83). Importantly, small chemical inhibitors of PI-273 that target the S-palmitoylated insertion and activation loop of human PI4KIIα demonstrate substantial suppression of breast cancer cell proliferation both *in vitro* and *in vivo (* 84). Flotillin-1 is a membrane-bound protein that plays a role in multicellular signaling processes within cells (85). Flotillin-1 is overexpressed in numerous solid tumors, and its S-palmitoylation enhances its stability and metastatic potential in breast cancer cells and experimental metastasis models (86, 87). Consequently, targeting the S-palmitoylation of flotillin-1 may represent a viable strategy to combat breast cancer metastasis (88). Another important factor in tumor metastasis is SMAD3, which, as a key protein molecule in the transforming growth factor-β (TGF-β) signaling pathway, plays a critical regulatory role in tumor metastasis (89). TGF-β plays an important role in cell growth, differentiation, immune regulation, and tumorigenesis and progression. When the TGF-β signaling pathway is activated, SMAD3 is phosphorylated and translocated to the nucleus, where it regulates the expression of genes related to tumor metastasis in conjunction with other transcription factors. TGF-β induces epithelial-mesenchymal transition (EMT), which promotes migration, invasion, and tumor cell metastasis, as well as suppressing the immune response by regulating immune cells in the tumor microenvironment (90). The S-palmitoylation of SMAD3, facilitated by ZDHHC19, enhances its activation. The interaction between SMAD3 and EP300 enhances the expression of mesenchymal markers associated with the mesenchymal subtype of glioblastoma multiforme (GBM). Consequently, inhibiting SMAD3 S-palmitoylation may be a pivotal molecular strategy to mitigate tumor spread (91).

## 5 Tumor immunotherapy

Before the 21st century, the primary modalities for cancer treatment included surgical resection, radiotherapy, chemotherapy, and targeted therapy. Cancer can theoretically be healed with the total excision of tumor tissue; however, many tumors metastasize before detection, and most surgical resections involve radical removal of the entire organ, resulting in significant patient harm, despite radiation therapy’s efficacy in eliminating the majority of tumor cells by high radiation doses, residual micrometastatic tumor cells persist, complicating total eradication (92). In the last twenty years, numerous therapies have been formulated based on research discoveries in immuno-oncology. Immunotherapy, designed to enhance the immune system’s capacity to eliminate malignant cells, significantly advances tumor treatment (93). Notwithstanding restricted response rates, prolonged clinical efficacy of immunotherapy has been evidenced across several cancer types (94–96). Numerous immunotherapies, such as immune checkpoint inhibitors (ICIs), cancer vaccines, adoptive cell transfer (ACT), and oncolytic virus therapy (OVT), have demonstrated promising outcomes; yet, each of these treatments in clinical practice possesses distinct limitations (97, 98).

Immune checkpoint compounds have garnered significant attention in tumor immunotherapy. PD-1 on T cells interacts with its ligands PD-L1/PD-L2 to provide inhibitory signals to T cells. This facilitates self-tolerance and enables cancer cells to evade immune destruction (99). Palmitoyltransferase ZDHHC3 has been documented to facilitate S-palmitoylation of PD-L1 at the cysteine Cys272 locus in colorectal cancer cells. PD-L1 undergoes S-palmitoylation in its cytoplasmic structural domain, stabilizing PD-L1 by preventing its ubiquitination and subsequent lysosomal degradation. Consequently, inhibiting PD-L1 palmitoylation enhances the cytotoxic T-cell-mediated destruction of cancer cells (100). ZDHHC9 has been identified as a palmitoyltransferase for PD-L1 in breast cancer cells. ZDHHC9 palmitoylates PD-L1, and this palmitoylation is crucial for its capacity to trigger mTOR signaling in cancer cells. mTOR is a protein kinase that plays a key role in cells and is involved in processes such as cell growth, proliferation, survival and metabolism. Although the PD-L1 signaling pathway does not directly regulate mTOR activity, PD-L1-mediated immunosuppression may affect the overall metabolic state and proliferative capacity of cells, thus indirectly affecting mTOR activity (101).

The binding of specific activating receptors can initiate the activation of NK cells. Natural killer group 2 member D (NKG2D) is a C-type lectin receptor that activates upon recognizing cell-surface MHC class I proteins, which are elevated in response to physiological stress. MICA and MICB (MHC-class I-associated chain A/B) serve as ligands for NKG2D, which activates the NK cell cytotoxic function by binding to the NKG2D receptors on NK cell surfaces, thus enabling the recognition and destruction of tumor cells that produce these molecules (102). Research indicates that MICA molecules experience S-palmitoylation at the Cys306 and Cys307 residues. Palmitoylation facilitates the surface expression of MICA and augments their interaction with the NK cell receptor NKG2D, therefore amplifying NK cell activation and tumor cell cytotoxicity (103). The S-palmitoylation status of MICA is crucial for tumor immunotherapy, and comprehensive research in this area is anticipated to yield novel approaches and targets for advancing future immunotherapeutic strategies.

## 6 The significance of S-palmitoylation in antitumor immunotherapy

Recent research indicates that modulating the palmitoylation process might boost or inhibit various immune cell functions, improving immunotherapeutic results (104). In the tumor microenvironment, regulatory T cells (Treg cells) facilitate carcinogenesis and progression by inhibiting effector cell function and enhancing tumor immune evasion through various pathways (105). Foxp3 is a particular Treg cell marker crucial for sustaining immunological tolerance and modulating autoimmune responses. It suppresses autoimmune and anti-tumor immune responses by modulating T cells’ activity and metabolic status (106). Foxp3 has been demonstrated to be S-palmitoylated, with its palmitoylation mediated by several members of the palmitoyltransferase ZDHHC family. Moreover, the inhibition of Foxp3 palmitoylation markedly diminished the intranuclear expression of Foxp3 in peripheral immune organs and tumor-infiltrating Treg cells, thereby impairing Treg cell function within the tumor microenvironment and enhancing the activation and efficacy of anti-tumor T cells (107).

### 6.1 S-palmitoylation influences sensitivity and resistance to tumor immunotherapy

Many oncogenic or oncostatic proteins involved in tumorigenesis and drug resistance are regulated by S-palmitoylation. Various immunosuppressive pathways have been identified in the cancer microenvironment (108), and mutations in IFN and MHC signaling genes result in resistance to immunotherapy. Patients with colorectal cancer infrequently display mutations in IFN and MHC signaling genes and typically demonstrate resistance to immunotherapy (109). Deficiency of optic nerve phosphatase has been shown to diminish IFNGR1 and MHC-I expression, compromising T-cell immunity (110). Reports indicate that IFNGR1 undergoes S-palmitoylation at Cys122, interacts with Adaptor Related Protein Complex 3 Subunit Delta 1(AP3D1), and is directed to lysosomes for destruction. Optic nerve phosphatase unexpectedly inhibits IFNGR1 degradation by binding to AP3D1, obstructing the recruitment of palmitoylated IFNGR1 to lysosomes, thus preserving the integrity of IFN-γ and MHC-I signaling (111). Consequently, pharmacological targeting of IFNGR1 palmitoylation stabilizes IFNGR1, augments T-cell immunity, and increases sensitivity to checkpoint treatment in colorectal cancer (111, 112).

Hyperimmune cell infiltration induced by glioblastoma (GBM) creates an immunosuppressive tumor microenvironment that fosters resistance to immunotherapy (113, 114). ZDHHCs, aberrantly produced in gliomas, may function via the phosphatidylinositol 3-kinase/protein kinase B (PI3K/AKT) signaling pathway. Inhibition of ZDHHCs by 2-bromopalmitate (2-BP) diminished glioma cell survival and autophagy while enhancing apoptosis (115, 116). Targeting ZDHHC promotes the sensitivity of glioma cells to temozolomide (TMZ) chemotherapy (117, 118).

Previous studies indicate a significant correlation between fatty acid synthase (FASN) expression and PD-L1 levels in cisplatin-resistant lung cancer cells and human T-cell leukemia lines (119, 120). The function of protein palmitoylation in conferring resistance to cisplatin-based systemic treatment in cancer remains unclear. Cisplatin-based systemic chemotherapy is presently the gold standard for treating metastatic bladder cancer (BC) (121–123), with no viable alternatives following the development of resistance. PD-L1 is extensively palmitoylated in drug-resistant cells (124, 125). The pharmacology of FASN suppressed the palmitoylation and expression of PD-L1, indicating a significant function for PD-L1 palmitoylation in conferring resistance to breast cancer treatment (126).

Sorafenib is a new multi-targeted antitumor drug, belonging to the multi-kinase inhibitors, which can exert anti-tumor effects by inhibiting multiple protein kinases and receptors (127). Sorafenib is now the first-line treatment for advanced hepatocellular carcinoma (HCC). Sorafenib is currently the primary treatment for advanced HCC. Aberrant activation of AKT signaling is a significant mechanism behind sorafenib resistance in patients with HCC (128, 129). The down-regulation of preprotein convertase Bacillus subtilis protease/kexin type 9 (PCSK9) augments tumor infiltration by cytotoxic T-cells, improves the effectiveness of anti-PD-1 therapies, and is pivotal in the anti-tumor immune response (130). The study indicates that abnormal overexpression of PCSK9 enhances cell proliferation and confers resistance to sorafenib in hepatocellular carcinoma by facilitating AKT-S473 phosphorylation (131). Palmitoylation of PCSK9 at the Cys600 site, facilitated by PCSK9 via ZDHHC16, triggers lysosomal-mediated degradation of PTEN and subsequent activation of AKT. Consequently, the inhibition of PCSK9 palmitoylation amplifies the anticancer efficacy of sorafenib in hepatocellular carcinoma (HCC) (132).

The majority of pancreatic cancer patients exhibit resistance to immune checkpoint-blocking therapies (ICBs) (133). Research indicates that ZDHHC9 is overexpressed in pancreatic cancer tissues and correlates with diminished antitumor immunity. The overexpression of ZDHHC9 in pancreatic tumors inhibits host antitumor immunity, suggesting that downregulating ZDHHC9 may be a practical immunotherapeutic approach with anti-PD-L1 therapy for pancreatic cancer (134).

### 6.2 Enhanced tumor immunotherapy with S-palmitoylation inhibitors

Protein S-palmitoylation is integral to cancer growth and antitumor immunity, rendering it a compelling target for cancer treatment. Palmitoylation inhibitors could be advantageous for cancer treatment, as numerous oncogenes necessitate S-palmitoylation alterations for appropriate location at the cell membrane (135). Recent research indicates that palmitoylation inhibitors regulates cancer-related pathways and influences tumorigenesis and progression, as shown in **Table 1**.

**Table 1:** Role of various palmitoylation inhibitors in tumor immunotherapy.

Acylprotein transferases (ZDHHCs)	Target protein	Cysteine site	Inhibitor	Effects of targeted palmitoylation on tumors	References
ZDHHC2/3/7	Foxp3	Cys204/218 C280/281	Knockdown ZDHHC2	Promoting the activation and effects of anti-tumor T cells	(103)
ZDHHC3	IFNGR1	Cys122	Cerulean	Enhanced sensitivity to immune checkpoint therapy for colorectal cancer	(111)
ZDHHC16	PCSK9	Cys600	GFP-PCSK9-PALM-1	Enhancing the antitumor effect of sorafenib in HCC	(132)
/	ZDHHC9	/	ZDHHC9-siRNA nanoparticles	Enhanced anti-PD-L1 therapy for pancreatic cancer	(134)
/	STING	Cys91	4-Octyl itaconate (4-OI)	Enhanced treatment of autoimmune diseases	(136, 137)
ZDHHC3	PD-L1	Cys272	Benzosceptrin C	Enhancement of tumor immunotherapy by the combination of Benzosceptrin C and anti-CTLA4	(138)
/	PPT1	/	DC661, Ezurpimtrostat	Enhancement of anti-tumor efficacy of anti-PD-1 antibody	(139, 140) (141)
LYPLAL1	cGAS	Cys404/405	LYPLAL1-IN-1/Knockdown LYPLAL1	Enhancement of cGAS-mediated immune response to improve PD-1 efficacy	(142)
ZDHHC5	NOD1/2	Cys558/567/952 Cys395/1033	2-BP	Enhancement of immune response and treatment of autoimmune diseases	(112)
ZDHHC3/21	FANS MHC-I	Cys1317	Orlistat and TVB-2640	Promotes antigen presentation and cytotoxicity of CD8+ T cells	(143, 144)

The cyclic guanosine monophosphate adenosine synthase (cGAS) and interferon gene-stimulating factor (STING) axis are essential for defending against invading infections and preserving immunological homeostasis. 4-Octyl itaconate (4-OI) was discovered to limit cGAS-STING activation by directly alkylating STING at Cys91, obstructing STING palmitoylation and oligomerization (136). It evidenced an interaction among various post-translational alterations of STING. This investigation indicated that 4-OI can reduce cGAS-STING-mediated autoimmune inflammation, offering a novel method for treating associated autoimmune diseases (137). Targeting the blockage of programmed cell death-1 (PD-1) and programmed cell death ligand-1 (PD-L1) has emerged as a cornerstone of cancer immunotherapy (145, 146). Benzosceptrin C was discovered to augment T-cell cytotoxicity towards cancer cells by reducing the levels of PD-L1. Benzosceptrin C may impede the palmitoylation of PD-L1 by obstructing the activity of the ZDHHC3 enzyme, hence initiating lysosome-mediated degradation of PD-L1. Consequently, the amalgamation of Benzosceptrin C and anti-CTLA4 significantly improves the effectiveness of cancer immunotherapy (138). Antitumor immunity is augmented when autophagy inhibition is synergized with immunotherapy (147–149). Treatment of cells with DC661, an inhibitor of palmitoyl protein thioesterase 1 (PPT1), activates naïve T cells and augments T-cell-mediated cytotoxicity, necessitating the expression of Calreticulin (CALR) proteins on the cell surface (139). This illustrates that lysosomal inhibition induces specific types of cell-intrinsic immunogenicity, suggesting a strategic combination of immunotherapy and lysosomal inhibition is promising (140). Inhibition of PPT1 by Ezurpimtrostat has been shown to decrease hepatic tumor burden in a murine model of hepatocellular carcinoma by promoting lymphocyte infiltration into tumors in conjunction with anti-programmed death-1 (PD-1) therapy. Inhibition of PPT1 augments anti-PD-1 immunotherapy by elevating major histocompatibility complex (MHC)-I expression on hepatocellular carcinoma cell surfaces and influences immunity via the recolonization and activation of cytotoxic CD8+ lymphocytes (141).

Aberrant activation of innate immune mechanisms correlates with several illnesses (150). Identification and characterization of a highly potent and selective minor molecule antagonist of the interferon gene-stimulating factor (STING) protein, which covalently targets the predicted transmembrane structure Cys91 to inhibit activation-induced palmitoylation of STING. This study illustrates that palmitoylation of STING is crucial for its assembly into multimeric complexes within the Golgi and for the recruitment of downstream signaling factors, thereby validating the potential of targeted STING therapies for treating autoinflammatory diseases (151). cGAS palmitoylation is prevalent in various tumor cell lines, and this modification is essential for cGAS to detect DNA and activate immunological signaling pathways (152). The LYPLAL1 protein induces cGAS depalmitoylation, hence diminishing the enzymatic activity of cGAS. Inhibition of LYPLAL1 markedly amplifies cGAS-mediated immune responses and augments the effectiveness of PD-1 inhibitors (142).

Immune checkpoint inhibitors (ICIs) have revolutionized the treatment paradigm of hepatocellular carcinoma (HCC). Regrettably, individuals exhibiting diminished MHC-I expression continue to be unresponsive to immune checkpoint inhibitors, and the display of cancer antigens through MHC-I (or HLA-I) is essential for initiating a robust anti-tumor immune response (153). Preclinical investigations have shown that the downregulation of MHC-I is a prevalent mechanism by which cancer cells avoid immune monitoring. Consequently, enhancing MHC-I expression is advantageous for facilitating cytotoxic T-cell-mediated apoptosis of cancer cells (147, 154, 155).

Palmitoyltransferase ZDHHC21 is a crucial regulator of fatty acid synthase (FASN) S-palmitoylation, interacting with FASN and facilitating its palmitoylation at Cys1317. This process results in diminished FASN protein stability and fatty acid synthesis, impeding the advancement of diffuse large B-cell lymphoma (143). Recent findings indicate that FASN inhibition diminishes S-palmitoylation of MHC-I, resulting in its lysosomal destruction. Palmitoyltransferase DHHC3 directly interacts with MHC-I and downregulates MHC-I protein levels. Consequently, the inhibition of FASN using orlistat and TVB2640, which obstructs MHC-I palmitoylation and lysosomal degradation to elevate its protein levels, further facilitates antigen presentation and CD8+ T cell cytotoxicity, hence augmenting the efficacy of immune checkpoint inhibition (144).

## 7 Conclusions and outlook

In recent years, tumor immunotherapy has evolved rapidly, promising results in some patients and offering potential for long-term treatment. However, current cancer immunotherapies still face challenges such as low response rates and the risk of serious immune-related adverse events, and the efficacy of tumor immunotherapy is mainly dependent on the tumor microenvironment (97). S-palmitoylation, an essential post-translational modification of proteins that plays a role in various aspects of tumor cell proliferation, apoptosis, and drug resistance, affects the tumor microenvironment by modulating the palmitoylation levels of specific proteins anti-tumor immune responses in the environment (156).

Inhibition of protein palmitoylation can be achieved, on the one hand, by developing specific inhibitors of ZDHHCs and, on the other hand, by designing peptide inhibitors that bind ZDHHCs competitively with substrate proteins (157). To date, no exact and potent inhibitors against ZDHHCs have been developed. However, considering that ZDHHCs may have multiple or even cross-substrates for palmitoylation, Potential inhibitors of ZDHHCs may face unintended consequences that may reduce their translational value in the clinic (158).

The most common S-palmitoylation inhibitor currently available is 2-BP, which may damage all ZDHHCs and react with other proteins. Therefore, it is necessary to resolve the structure of ZDHHCs to develop selective inhibitors of ZDHHCs. However, because the catalytic structural domains of ZDHHC family members are very similar, the development of specific inhibitors of ZDHHCs is very challenging, and targeting the C-terminus and N-terminus of ZDHHCs with sequence diversity may be an effective strategy for designing specific ZDHHCs inhibitors in the future. Since each ZDHHC acts on various substrates, the impact of explicitly inhibiting the function of a particular ZDHHC may be multifaceted and needs to be explored in further studies.

Palmitoylation is an emerging target for tumor immunotherapeutic drugs with great potential and broad application prospects. With the deepening research on the mechanism of palmitoylation and the emergence of novel inhibitors, it is believed that more tumor immunotherapeutic drugs targeting palmitoylation will be introduced, bringing new therapeutic choices and hope to cancer patients.
